# Long-term depression at hippocampal mossy fiber-CA3 synapses involves BDNF but is not mediated by p75NTR signaling

**DOI:** 10.1038/s41598-021-87769-9

**Published:** 2021-04-20

**Authors:** Machhindra Garad, Elke Edelmann, Volkmar Leßmann

**Affiliations:** 1 Institute of Physiology, Otto-von-Guericke University Magdeburg, 39120 Magdeburg, Germany; 2 Center for Behavioral Brain Sciences (CBBS), Magdeburg, Germany

**Keywords:** Cellular neuroscience, Neuronal physiology, Neurotrophic factors, Synaptic plasticity, Synaptic transmission

## Abstract

BDNF plays a crucial role in the regulation of synaptic plasticity. It is synthesized as a precursor (proBDNF) that can be proteolytically cleaved to mature BDNF (mBDNF). Previous studies revealed a bidirectional mode of BDNF actions, where long-term potentiation (LTP) was mediated by mBDNF through tropomyosin related kinase (Trk) B receptors whereas long-term depression (LTD) depended on proBDNF/p75 neurotrophin receptor (p75NTR) signaling. While most experimental evidence for this BDNF dependence of synaptic plasticity in the hippocampus was derived from Schaffer collateral (SC)-CA1 synapses, much less is known about the mechanisms of synaptic plasticity, in particular LTD, at hippocampal mossy fiber (MF) synapses onto CA3 neurons. Since proBDNF and mBDNF are expressed most abundantly at MF-CA3 synapses in the rodent brain and we had shown previously that MF-LTP depends on mBDNF/TrkB signaling, we now explored the role of proBDNF/p75NTR signaling in MF-LTD. Our results show that neither acute nor chronic inhibition of p75NTR signaling impairs MF-LTD, while short-term plasticity, in particular paired-pulse facilitation, at MF-CA3 synapses is affected by a lack of functional p75NTR signaling. Furthermore, MF-CA3 synapses showed normal LTD upon acute inhibition of TrkB receptor signaling. Nonetheless, acute inhibition of plasminogen activator inhibitor-1 (PAI-1), an inhibitor of both intracellular and extracellular proBDNF cleavage, impaired MF-LTD. This seems to indicate that LTD at MF-CA3 synapses involves BDNF, however, MF-LTD does not depend on p75NTRs. Altogether, our experiments demonstrate that p75NTR signaling is not warranted for all glutamatergic synapses but rather needs to be checked separately for every synaptic connection.

## Introduction

Brain-derived neurotrophic factor (BDNF) is synthesized as a precursor (proBDNF) in the endoplasmic reticulum that can be proteolytically cleaved and thereby converted into mature BDNF (mBDNF)^1–3^. Cleavage of proBDNF can take place either intracellularly in the Golgi or in secretory vesicles by furin or proprotein convertases^4^ or through extracellular action of matrix metalloproteinases or components of the tissue plasminogen activator/plasmin (tPA/plasmin) proteolytic cascade^5,6^. Plasminogen activator inhibitor-1 (PAI-1) inhibits tPA and furin, thereby inhibiting both extracellular and intracellular proBDNF cleavage^7,8^. ProBDNF primarily binds to p75 neurotrophin receptors (p75NTR), but it can also bind to tropomyosin related kinase (Trk) B receptors^5,9,10^. In contrast, mBDNF binds to and activates TrkB receptors with high affinity, and p75NTR with low affinity^11–13^. Results from several studies support a yin-yang mechanism, where proBDNF and mBDNF elicit opposite biological outcomes through the activation of p75NTR and TrkB receptors, respectively^1,14–16^. At hippocampal Schaffer collateral (SC)- Cornu Ammonis (CA) 1 synapses, proBDNF promotes long-term depression (LTD) by activating p75NTR^17,18^, whereas mBDNF/TrkB signaling is necessary for long-term potentiation (LTP)^19–23^.

The main input to the hippocampus is provided by the dentate gyrus^24^. Dentate granule cell axons send information to the CA3 region through mossy fiber (MF) synapses^25^. MF-CA3 synapses show three important unique features: low basal presynaptic release probability, pronounced paired-pulse facilitation, and frequency facilitation^24^. In addition to the prominent form of *N*-methyl-d-aspartate receptor (NMDAR) independent long-term synaptic plasticity, MF synapses can also express NMDAR-dependent long-term synaptic plasticity^24,26^. From a functional perspective, the dentate gyrus granule cells and CA3 pyramidal cells play a crucial role in pattern separation and pattern completion^27,28^. This suggests that granule cells and CA3 neurons are key to memory formation, a hypothesis that can be investigated at the cellular level by assessing synaptic plasticity. Furthermore, all principal cells in the hippocampus—the granule cells in the dentate gyrus, the pyramidal neurons in CA3 and CA1—express BDNF^29,30^. Yang and coworkers (2009) reported that uncleaved proBDNF is expressed at substantial levels in the adult brain, including the hippocampal MF termination zone^31^. Moreover, MF terminals on CA3 pyramidal cells are highly enriched in both proBDNF and mBDNF^31–33^. Earlier, our group demonstrated that acute and chronic mBDNF/TrkB signaling is crucial for MF-LTP in adult mice^34^, indicating that even the NMDAR independent MF-LTP employs mBDNF-dependent increase in synaptic transmission. Furthermore, BDNF and cAMP modulatory effects were shown for immature MF-CA3 synapses^35^. In general, however, little is known about MF-LTD and the role of proBDNF/p75NTR signaling in this process.

Using field potential recordings at hippocampal MF-CA3 synapses in acute slices obtained from 8 to 14 weeks old C57Bl/6J mice, we observed that acute inhibition or challenging of p75NTR signaling, either by the p75NTR inhibitor TAT-Pep5^36^ or by including the p75NTR ligand LM11A31^37^ in the bath solution, did not change the magnitude of MF-LTD induced by standard low frequency stimulation (LFS; 1 Hz, 15 min) or paired-pulse LFS (inter-pulse interval 50 ms; 1 Hz, 15 min)^38,39^. Likewise, chronic absence of p75 receptor signaling in p75NTR^ExIV^ knock-out mice^40^ also did not result in impaired MF-LTD. Interestingly, acute inhibition of plasminogen activator inhibitor-1 (PAI-1) by tiplaxtinin^41^, a manipulation which leads to reduced extracellular proBDNF and increased mBDNF levels, significantly impaired MF-LTD. Acute inhibition of TrkB signaling with the Trk kinase inhibitor K252a^34,35,42^ or ANA-12, a selective TrkB receptor inhibitor^43^, did not affect MF-LTD. These results suggest that the induction and/or expression of MF-LTD involves BDNF but is not mediated by p75NTR signaling. This indicates that LTD at SC-CA1 but not at MF-CA3 synapses depends on p75NTR signaling.

## Results

Synaptic plasticity at Mossy fiber (MF)—Cornu Ammonis (CA) 3 synapses shows unique features in short-term effects and expression pattern^24^. In the current study, we asked whether the pro-form of brain-derived neurotrophic factor (BDNF), i.e. proBDNF and its signaling through p75 neurotrophin receptors (p75NTR) is involved in long-term depression (LTD) at hippocampal MF-CA3 synapses.

### MF-CA3 and associational/commissural fiber (A/C)-CA3 synapses show different basal synaptic transmission and short-term plasticity but comparable LTD

Since MF signals are often obscured by overlaid signals from A/C synapses, we assured in a first series of experiments that we can clearly distinguish between both synaptic inputs to CA3 neurons. Our results indicated that under our recording conditions we can differentiate pure MF signals from AC fiber signals^34^ (compare Supplementary Fig. S1), forming the basis for our approach to answer whether proBDNF/p75NTR signaling is involved in LTD at MF-CA3 synapses.

**Figure 1:**
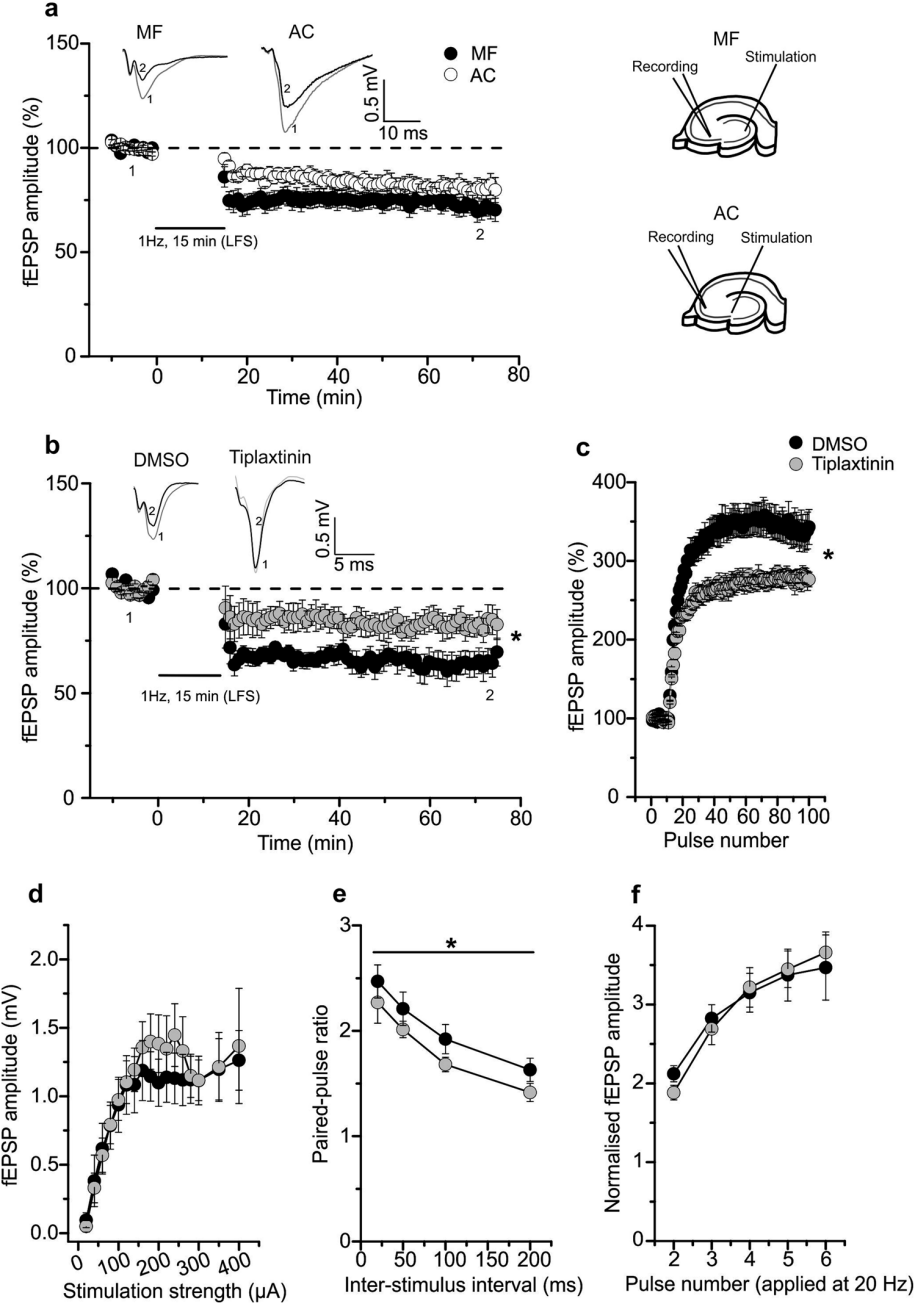
Mossy fiber (MF)-CA3 synapses show BDNF-dependent LTD, frequency facilitation and paired-pulse facilitation. **(a)** LTD magnitude at MF-CA3 synapses induced by low frequency stimulation (LFS, 900 pulses, 1 Hz) was similar to AC synapses (filled circle: MF (n = 16/N = 12); open circle: AC fibers (n = 10/N = 7)). Representative averaged original responses are shown for both, MF and AC fiber LTD. In the graph, “1” depicts mean fEPSP amplitudes of first 10 min of baseline and “2” indicates mean fEPSP amplitudes between 55 and 60 min after induction of LTD. (**b**) LTD at MF-CA3 synapses is significantly impaired in the presence of tiplaxtinin (150 µM; an inhibitor of plasminogen activator inhibitor-1) in comparison to DMSO treated control (filled circle: DMSO (n = 8/N = 6); filled gray circle:Tiplaxtinin (n = 8/N = 6). The inset shows representative averaged original fEPSP responses. For MF-LTD in DMSO and in the presence of tiplaxtinin, “1” depicts mean fEPSP amplitudes of first 10 min of baseline and “2” depict mean fEPSP amplitudes between 55 and 60 min after induction of LTD. (**c**) and (**e**) Tiplaxtinin treated slices showed significantly decreased frequency facilitation (**c**) and paired-pulse facilitation (**e**) at MF-CA3 synapses (filled circle: DMSO (n = 9/N = 7); filled gray circle:Tiplaxtinin (n = 9/N = 7). (**d**) and (**f**) Input–output curve of basal synaptic responses (c; filled circle: DMSO (n = 7/N = 5); filled gray circle: Tiplaxtinin (n = 9/N = 7)) and train facilitation (e; filled circle: DMSO (n = 9/N = 7); filled gray circle: Tiplaxtinin (n = 9/N = 7)) at MF synapses exhibited no change in presence of tiplaxtinin compared to DMSO control. Data shown as mean ± SEM. Scale bars are exhibited in the inset. *p < 0.05 (two-tailed Student’s t-test, Mann–Whitney U-test or ANOVA).

Next, we tested both, MF and A/C pathways, for the capability to undergo LTD by low frequency stimulation (LFS). We observed successful induction and maintenance of LTD in MF as well as A/C pathways. Interestingly both forms of LTD, i.e. MF-LTD and A/C-LTD in the CA3 region, presented similar magnitudes and time courses of LTD induced by a 15 min stimulation at 1 Hz (Fig. 1a; MF-CA3 LTD: 70.9 ± 4.8% (n = 16/N = 12); A/C LTD: 80.6 ± 4.9% (n = 10/N = 7), Mann–Whitney U-test U = 99.0, df = 1, p = 0.317). As negative control to test the stability of our recording conditions, we measured MF fEPSPs without applying an LTD induction protocol and observed stable fEPSP amplitudes for > 90 min (Figs. S5a, S5c; 97.2 ± 3.6% (n = 9/N = 6), 1-sample Student’s t-test t_(8)_ = 0.791 p = 0.4517). Moreover, we did not observe a change in field potential amplitudes at MF-CA3 and AC-CA3 synapses in slices that had been subjected to short-term plasticity paradigms (i.e., train facilitation, frequency facilitation; Fig. S5b; MF: 93.3 ± 4.2% (n = 12/N = 7), 1-sample Student’s t-test t_(11)_ = 1.581 p = 0.1423; Fig. S5d; AC: 96.1 ± 2.4% (n = 10/ N = 7), 1-sample Student’s t-test t_(9)_ = 1.5986 p = 0.1444). These results indicate the stability of our recording conditions for over 90 min, the time period LTD was recorded for in the present study, and that the short-term plasticity paradigms used in our study did not change the basal field potential amplitudes.

Next, we determined whether activation of N-methyl D-aspartate receptors (NMDARs) is involved in MF-LTD. To this aim, 50 µM APV was applied to the recording chamber starting > 1 h before induction of LTD. Similar to previous results for long-term potentiation (LTP) at MF synapses^34,44,45^, we observed that also MF-LTD was independent of NMDAR activation (Fig. S2; MF-LTD: 70.9 ± 4.8% (n = 16/N = 12); MF-LTD in APV: 76.2 ± 4.5% (n = 17/N = 13), two-tailed Student’s t-test t_(31)_ = − 0.8092 p = 0.4245).

To test for a possible role of NMDA receptors in regulating other aspects of MF-CA3 signaling besides LTD, we also investigated whether basal synaptic transmission and short-term plasticity at MF-CA3 synapses were affected by acute inhibition of NMDA receptors with APV (50 µM; pre-incubation > 1 h). While we observed a slightly steeper IO curve, PPF, train facilitation and frequency facilitation at MF-CA3 synapses were not affected in presence of APV (see supplementary results and Fig. S4).

### MF-CA3 synapses show BDNF-dependent LTD, frequency facilitation and PPF

To test whether proBDNF is involved in MF-LTD, we bath applied an inhibitor of the plasminogen activator inhibitor-1 (PAI-1; tiplaxtinin^41^ (150 µM in 0.1% DMSO), starting > 1 h before LTD induction, which disinhibits conversion of proBDNF to mature BDNF. Interestingly, LTD at MF-CA3 synapses was significantly impaired in the presence of Tiplaxtinin (Fig. 1b; DMSO: 64.4 ± 6.3% (n = 8/N = 6); Tiplaxtinin: 83.1 ± 6.0% (n = 8/N = 6), two-tailed Student’s t-test t_(14)_ = − 2.1589 p = 0.0486). In addition, Tiplaxtinin treated slices showed significantly decreased frequency facilitation (Fig. 1c; DMSO: 337.6 ± 21.4% (n = 9/N = 7); Tiplaxtinin: 279.1 ± 9.7% (n = 9/N = 7), Mann–Whitney U-test U = 64.0, df = 1, p = 0.038), and significantly reduced PPF at MF-CA3 synapses (Fig. 1e; PPF at 20 ms: DMSO: 2.5 ± 0.16 (n = 9/N = 7); Tiplaxtinin: 2.3 ± 0.19 (n = 9/N = 7), One-way ANOVA F_(1,63)_ = 5.127 p = 0.027) compared to solvent controls, whereas IO curves (Fig. 1d; half maximal stimulation; 200 µA: DMSO: 1.1 ± 0.17 (n = 7/N = 5); Tiplaxtinin: 1.4 ± 0.2 (n = 9/N = 7), One-way ANOVA F_(1,205)_ = 2.144 p = 0.1447) and train facilitation remained intact (Fig. 1f; TF at pulse 4: DMSO: 3.1 ± 0.25 (n = 9/N = 7); Tiplaxtinin: 3.2 ± 0.25 (n = 9/N = 7), One-way ANOVA F_(1,80)_ = 0.0023 p = 0.9617). These results suggest that proBDNF and/or mature BDNF is involved in the regulation of LTD and short-term plasticity, i.e. frequency facilitation and PPF, at MF-CA3 synapses in the hippocampus.

### LTD at MF-CA3 synapses is not mediated via p75NTR signaling

**Figure 2:**
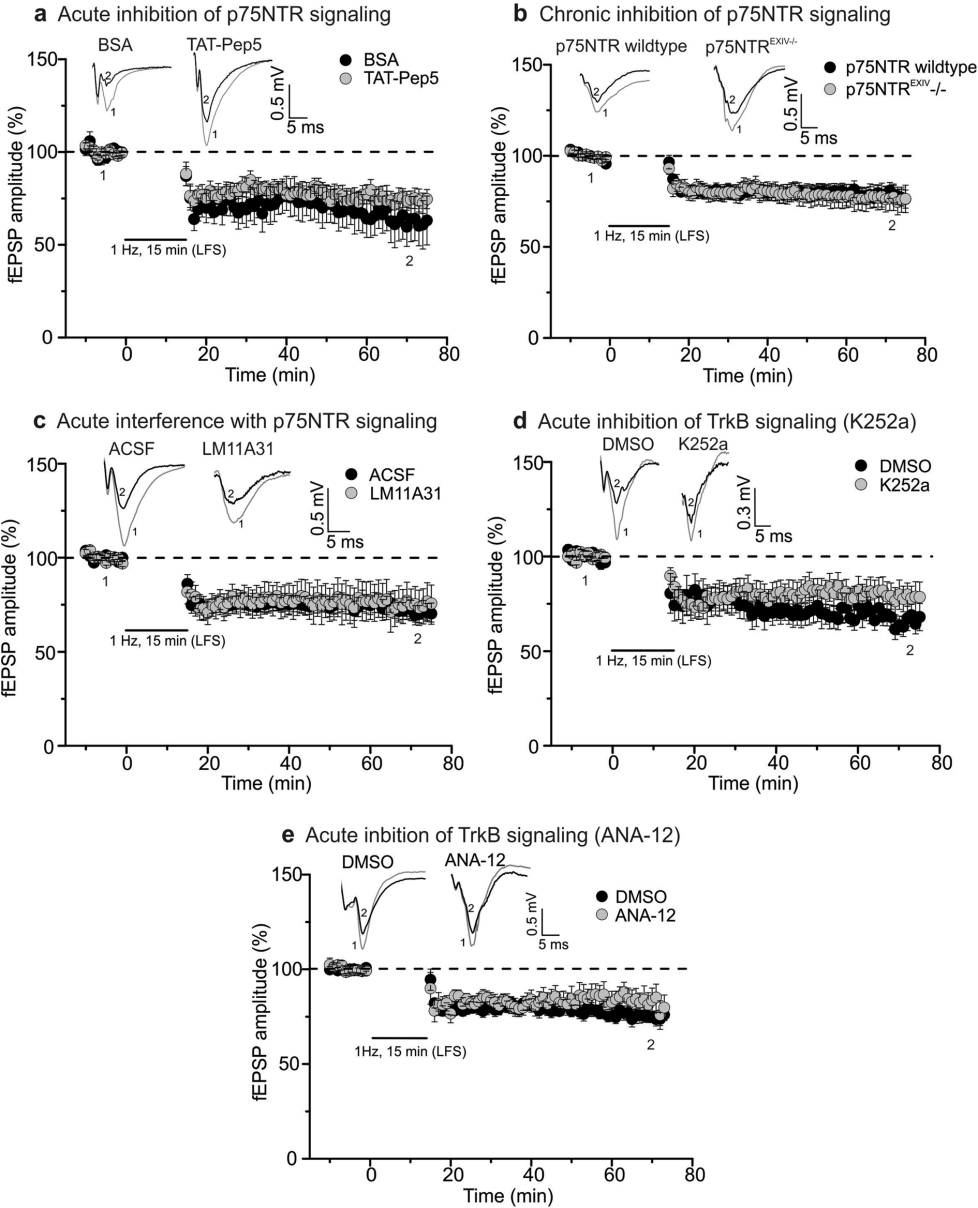
LTD at MF synapses is not mediated via p75NTR or TrkB receptor signaling. (**a**), (**c**) and (**d**) MF LTD is not impaired in the presence of bath applied TAT-Pep5 (1 µM, **a**), LM11A31 (100 nM, **c**) or K252a (200 nM, **d**) in comparison to the respective solvent controls (filled circle: BSA (**a**, n = 8/N = 6), ACSF (**c**, n = 16/N = 12) and DMSO (**d**, n = 12/N = 6); filled gray circle: TAT-Pep5 (**a**, n = 11/N = 10), LM11A31 (**c**, n = 11/N = 6) and K252a (**d**, n = 9/N = 5)). **b)** Recordings in slices of p75NTR^EXIV−/−^ mice displayed no influence of p75NTR chronic deficiency on LTD magnitudes at MF synapses (filled circle: p75NTR wildtype (n = 8/N = 5); filled gray circle: p75NTR^EXIV−/−^ (n = 12/N = 7)). (**e**) Unaltered MF LTD in the presence of bath applied ANA-12 (20 µM), in comparison to the respective solvent control (filled circle: DMSO (n = 16/N = 11); filled gray circle: ANA-12 (**e**, n = 11/N = 6). The figure insets show representative averaged original fEPSP traces. For all MF-LTD experiments, “1” indicates mean fEPSP amplitudes of first 10 min of baseline and “2” depicts mean fEPSP amplitudes between 55 and 60 min after induction of LTD. Data expressed as mean ± SEM. Corresponding scale bars are shown as insets.

In the next set of experiments, we addressed the question whether proBDNF mediates MF-LTD through p75NTR signaling as described for LTD in CA1 region of the hippocampus^18,46^. In a first approach, we bath applied 1 µM of the p75NTR signaling inhibitor TAT-Pep5 (in 0.1% Bovine Serum Albumin (BSA)^36^, starting > 1 h before LTD induction. MF-LTD did not significantly change in magnitude or time course in the presence of TAT-Pep5, compared to the BSA control (Fig. 2a; BSA: 62.1 ± 11.6% (n = 8/N = 6), TAT-Pep5: 73.7 ± 5.2% (n = 11/N = 10), two-tailed Student’s t-test t_(17)_ = − 1.0129 p = 0.3253). As a positive control for the drug, we tested whether TAT-Pep5 was able to inhibit LTD at hippocampal SC-CA1 synapses. In agreement with previous results^17,18^, these experiments revealed significantly impaired LTD at Schaffer collateral (SC)-CA1 synapses in the presence of TAT-Pep5 compared to BSA control (Fig. S3; BSA: 68.1 ± 5.8% (n = 12/N = 8); TAT-Pep5: 83.8 ± 4.2% (n = 11/N = 7), two-tailed Student’s t-test t_(21)_ = − 2.1682 p = 0.0418), thus proving intact activity of TAT-Pep5 in our experiments.

Since this acute interference with p75NTR signaling did not impair MF-LTD, we next asked whether chronic deletion of p75NTR signaling would result in impaired MF-LTD. For these experiments we selected the p75NTR^ExIV^ knock-out (−/−) mouse line, which was shown previously to result in knock-out of truncated and non-truncated p75NTRs throughout the brain^40^. These homozygous p75NTR^EXIV−/−^ mice did also not show any impairment of MF-LTD in comparison to their wildtype littermates (Fig. 2b; WT: 76.0 ± 6.0% (n = 8/N = 5), p75NTR^EXIV−/−^: 78.3 ± 6.9% (n = 12/N = 7), Mann–Whitney U-test U = 39.0, df = 1, p = 0.487).

As an additional way to test for a possible role of p75NTRs in MF-LTD, we incubated our slices with the non-peptide low molecular weight p75NTR ligand LM11A31 (100 nM, dissolved in water), which was reported previously to interfere with p75NTR signaling at SC-CA1 synapses^37^. Since it was reported in this previous study that LM11A31 might exert either agonistic or antagonistic actions on p75NTRs, we tested whether the drug could modulate MF-LTD either positively or negatively. However, bath application of LM11A31 starting > 1 h prior to inducing LTD did neither enhance nor reduce MF-LTD (Fig. 2c; Ctrl: 70.9 ± 4.8% (n = 16/N = 12), LM11A31: 74.9 ± 9.3% (n = 11/N = 6), two-tailed Student’s t-test t_(25)_ = − 0.4198 p = 0.6782).

Whereas the former experiments indicated that initiation of MF-LTD occurred independent from p75NTR signaling, we next asked whether tropomyosin related kinase B (TrkB) receptor signaling is involved in MF-LTD. Importantly, although mBDNF is regarded as the main ligand of TrkB receptors^15,19^, also proBDNF was previously shown to activate TrkB receptors under certain conditions^9^. Thus, to test whether TrkB receptor signaling, upon activation by mBDNF and/or proBDNF, plays a role in MF-LTD, we bath applied the non-selective tyrosine kinase inhibitor K252a (200 nM in 0.1% DMSO) starting > 1 h prior to induction of LTD to the recorded hippocampal slices. Also under these conditions, MF-LTD was not significantly changed (Fig. 2d; DMSO: 64.4 ± 5.1% (n = 12/N = 6); K252a: 77.9 ± 6.7% (n = 9/N = 5), two-tailed Student’s t-test t_(19)_ = − 1.6389 p = 0.1177), thus speaking against a contribution of TrkB signaling in MF-LTD. To further investigate a possible involvement of TrkB signaling in MF-LTD, we used in another series of experiments bath application of the selective TrkB receptor inhibitor ANA-12 (20 µM in 0.1% DMSO), starting > 1 h prior to induction of LTD to the recorded hippocampal slices. In accordance with the results obtained with K252a, also ANA-12 did not significantly reduce MF-LTD (Fig. 2e; DMSO: 75.2 ± 3.1% (n = 16/N = 11); ANA-12: 82.9 ± 6.3% (n = 11/N = 6), two-tailed Student’s t-test t_(25)_ = − 1.2562 p = 0.1563), thus speaking against a contribution of TrkB receptor signaling in MF-LTD.

### Paired-pulse (pp) LFS induced LTD at hippocampal MF-CA3 synapses is also independent from p75NTR signaling

**Figure 3:**
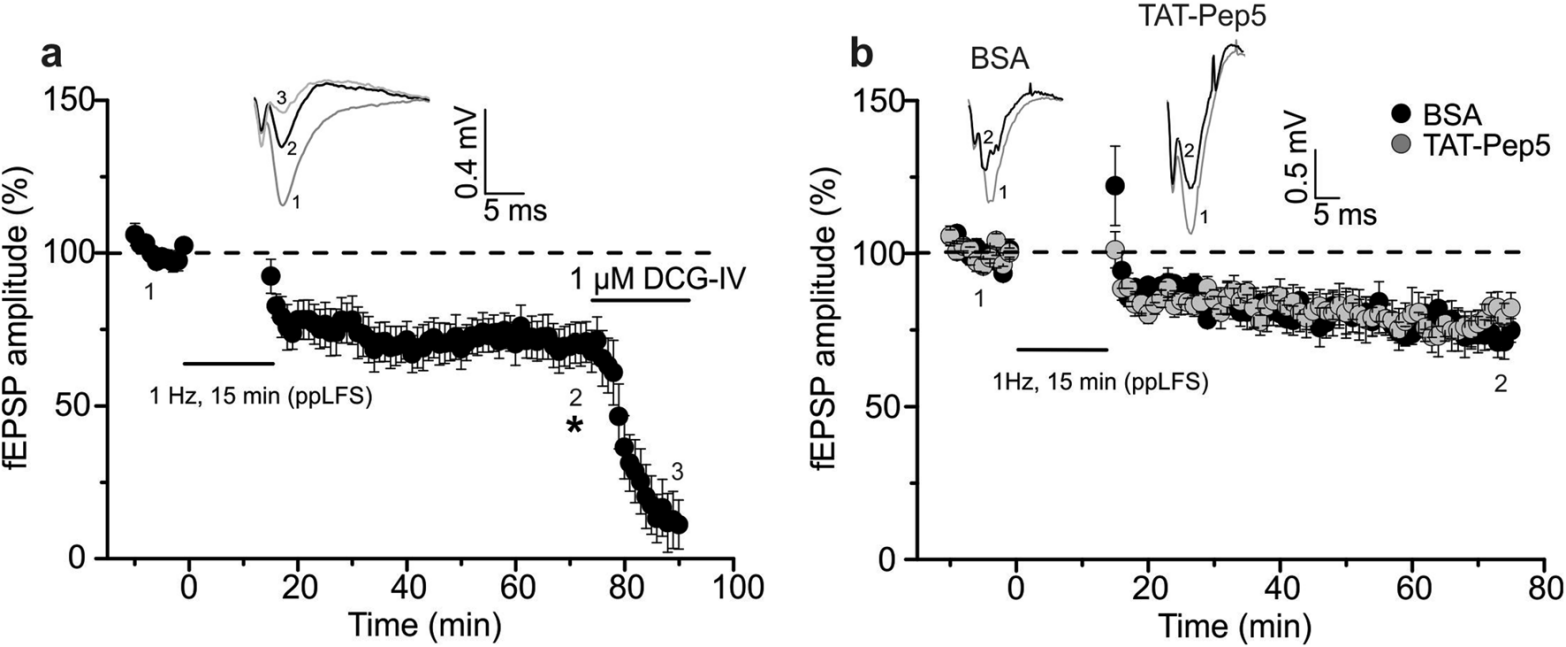
Paired-pulse LFS induced LTD at MF-CA3 synapses is also independent of p75NTR signaling. (**a**) Paired-pulse (pp) LFS induced significant LTD at MF synapses (filled circle: ppLFS (n = 10/N = 7)). The inset shows representative averaged original fEPSP responses. For MF-LTD, “1” depicts mean fEPSP amplitudes of first 10 min of baseline, “2” depict mean fEPSP amplitudes between 55 and 60 min after induction of LTD, and “3” shows mean fEPSP amplitude after DCG-IV incubation to verify pure MF origin. (**b**) MF-CA3 synapses exhibited comparable LTD magnitude in presence of TAT-Pep5 (1 µM; p75NTR inhibitor) compared to BSA control (filled circle: BSA (n = 8/N = 5); filled gray circle: TAT-Pep5 (n = 10/N = 5)). The inset shows representative averaged original fEPSP responses. For MF-LTD in BSA and in the presence of TAT-Pep5, “1” depicts mean fEPSP amplitudes of first 10 min of baseline and “2” depict mean fEPSP amplitudes between 55 and 60 min after induction of LTD. Data shown as mean ± SEM. Corresponding scale bars are shown as insets. *p < 0.05 (1-sample Student’s t-test or two-tailed Student’s t-test).

To test whether LTD induced by another induction paradigm does recruit p75NTR signaling, we studied ppLFS induced LTD^38,39^ at MF-CA3 synapses in the presence of TAT-Pep5. PpLFS induced significant LTD at MF-CA3 synapses, similar in magnitude and time course to our standard LFS (Fig. 3a; ppLFS: 69.7 ± 6.7% (n = 10/N = 7), 1-sample Student’s t-test t_(9)_ = 4.499 p = 0.0015; compare Fig. 1a). Next, we bath applied 1 µM of the p75NTR signaling inhibitor TAT-Pep5 (in 0.1% BSA), starting >1 h before LTD induction with ppLFS. However, also in this case MF-LTD did not significantly change in magnitude or time course in the presence of TAT-Pep5, compared to the BSA control (Fig. 3b; BSA: 72.4 ± 5.0% (n = 8/N = 5), TAT-Pep5: 80.1 ± 4.5% (n = 10/N = 5), two-tailed Student’s t-test t_(16)_ = − 1.1375 p = 0.2721).

Altogether, these results are consistent with a role of proBDNF in MF-LTD while p75NTR receptor signaling is not involved in this process, which is at variance with the proBDNF/p75NTR-dependent LTD described at hippocampal SC-CA1 synapses.

### p75NTR signaling plays a role in paired-pulse facilitation at MF-CA3 synapses

**Figure 4:**
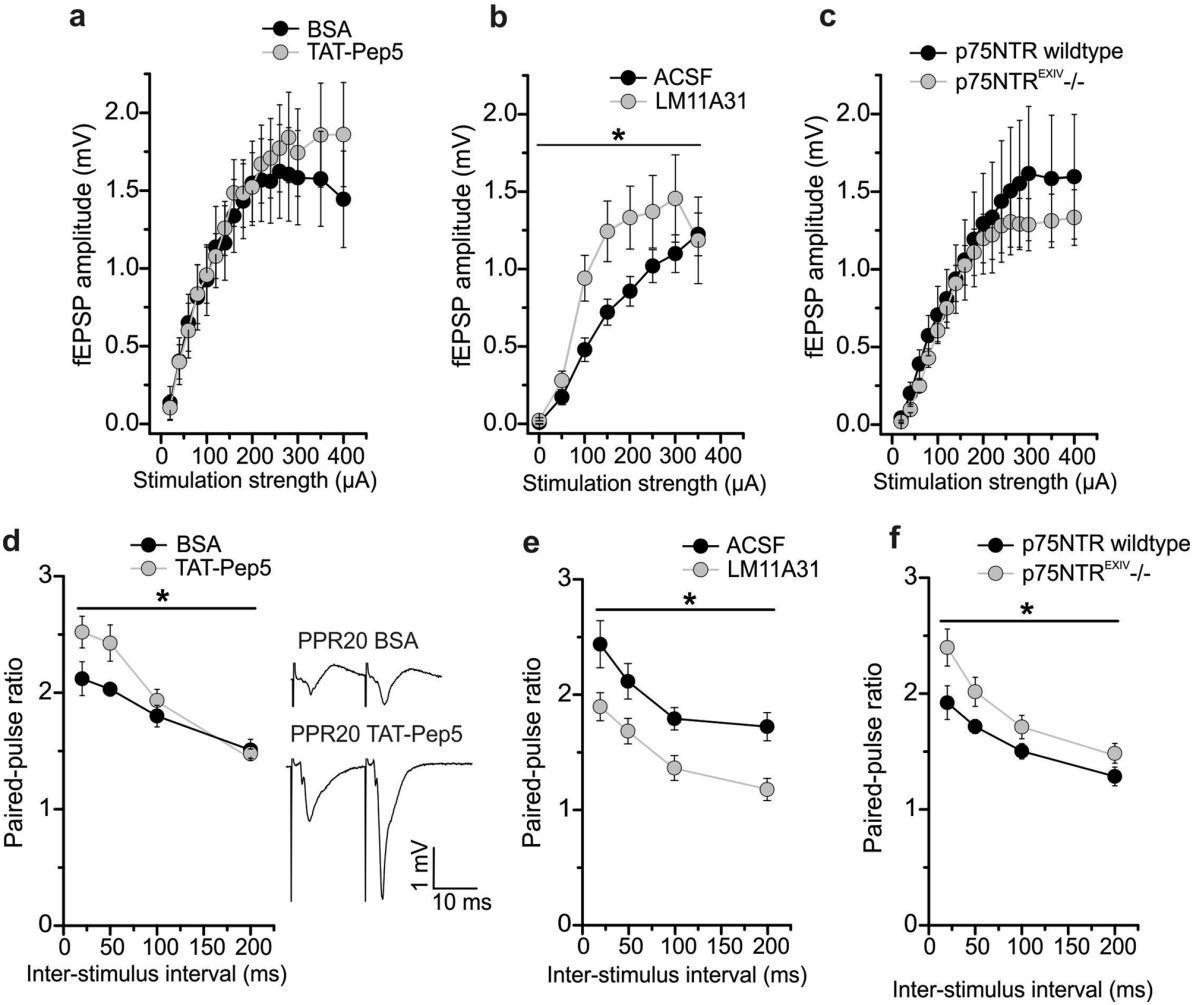
Influence of acute or chronic inhibition of p75NTR signaling on basal synaptic responses and short-term synaptic plasticity at hippocampal mossy fiber synapses. (**a**) and (**b**) input–output (IO) curve in the presence of 1 µM TAT-Pep5 (**a**) or 100 nM LM11A31 (**b**) for acute inhibition or modulation of MF-transmission. (**c**) IO curve in p75NTR^EXIV−/−^ mice (chronic inhibition). (**d**) and (**e**) paired-pulse facilitation (PPF) at different inter-stimulus intervals of (20, 50, 100 and 200 ms) in the presence of TAT-Pep5 (**d**) or LM11A31 (**e**). The inset displays representative averaged original responses for PPF at an ISI of 20 ms for BSA and TAT-Pep5. Scale bars are shown in the inset. (**f**) PPF in p75NTR^EXIV−/−^ mice. Data expressed as mean ± SEM. *p < 0.05 (ANOVA).

To test for a possible role of p75NTR signaling in regulating other aspects of MF-CA3 signaling besides LTD, we next investigated whether basal synaptic transmission and short-term plasticity at MF-CA3 synapses were affected after > 1 h pre-incubation of slices with the different p75NTR inhibitors. In the presence of TAT-Pep5, the IO curve of MF synapses exhibited no change compared to BSA treated control slices (Fig. 4a; half maximal stimulation intensity; 200 µA: BSA: 1.5 ± 0.27 mV (n = 11/N = 4), TAT-Pep5: 1.5 ± 0.22 mV (n = 9/N = 7), One-way ANOVA F_(1,272)_ = 1.127 p = 0.2894). The LM11A31 treated slices showed steeper IO curves compared to solvent control (Fig. 4b; half maximal stimulation intensity: 150 µA: Ctrl: 0.7 ± 0.08 mV (n = 16/N = 11), LM11A31: 1.2 ± 0.19 mV (n = 13/N = 8), One-way ANOVA F_(1,203)_ = 17.0 p < 0.0001). However, we observed significantly higher fEPSP amplitudes at MF-CA3 synapses only at 150 µA stimulation strength in LM11A31 treated slices compared to ACSF control (Bonferroni post-tests p = 0.0404). Since either agonistic or antagonistic functions on p75NTRs have been attributed to LM11A31, these results cannot be directly compared with TAT-Pep5 experiments. Moreover, MF-CA3 synapses in slices obtained from p75NTR^EXIV−/−^ mice displayed no change in the IO curve compared to slices from wildtype littermates (Fig. 4c; half maximal stimulation intensity; 200 µA: WT: 1.3 ± 0.32 mV (n = 10/N = 5), p75NTR^EXIV−/−^: 1.2 ± 0.16 mV (n = 18/N = 7), One-way ANOVA F_(1,404)_ = 2.589 p = 0.1084). Altogether, this indicates that neither acute (TAT-Pep5) nor chronic interference with p75NTR signaling influenced basal synaptic transmission at MF-CA3 synapses.

To get further insight into a possible role of p75NTR signaling in basal synaptic transmission and short-term synaptic plasticity, we tested PPF (expressed as paired pulse ratio, PPR) at different ISIs. PPF at MF-CA3 synapses was changed in TAT-Pep5 treated slices compared to BSA control (Fig. 4d; PPF at ISI 20 ms: BSA: 2.1 ± 0.14 (n = 11/N = 4), TAT-Pep5: 2.5 ± 0.14 (n = 11/N = 9), One-way ANOVA F_(1,79)_ = 8.464 p = 0.0047). PPR at ISI of 20 ms was significantly increased (Bonferroni post-test p = 0.044) in the presence of TAT-Pep5, while PPR at ISIs of 50, 100 and 200 ms exhibited no significant change. In the presence of LM11A31, MF synapses showed reduced PPF (Fig. 4e; PPF at ISI 20 ms: Ctrl: 2.4 ± 0.2 (n = 15/N = 10), LM11A31: 1.9 ± 0.12 (n = 12/N = 7), One-way ANOVA F_(1,100)_ = 25.0 p < 0.0001). Bonferroni post-tests revealed significantly decreased PPR at ISI of 20 ms (p = 0.0255) and 200 ms (p = 0.0246) in LM11A31 treated slices, while PPR at ISIs 50 and 100 ms exhibited no significant change. Again, since agonistic and antagonistic functions on p75NTRs were described for LM11A31, these results cannot be directly compared with the respective TAT-Pep5 experiments. Similar to the results obtained with TAT-Pep5, p75NTR^EXIV−/−^ mice also showed increased PPF (Fig. 4f; PPF at ISI 20 ms: WT: 1.9 ± 0.14 (n = 11/N = 5), p75NTR^EXIV−/−^: 2.4 ± 0.16 (n = 18/N = 7), One-way ANOVA F_(1,100)_ = 11.49 p = 0.001). PPR at an ISI of 20 ms was significantly increased (Bonferroni post-tests p = 0.0297) in p75NTR^EXIV−/−^ mice, while PPF at 50, 100 and 200 ms exhibited no statistically significant change. Taken together, acute (TAT-Pep5 results only) and chronic p75NTR interference (results in p75NTR^EXIV−/−^ mice) showed significant effects on PPF selectively occurring at 20 ms ISI.

### Hippocampal MF-CA3 synapses show intact train facilitation and frequency facilitation following acute or chronic inhibition of p75NTR signaling

**Figure 5:**
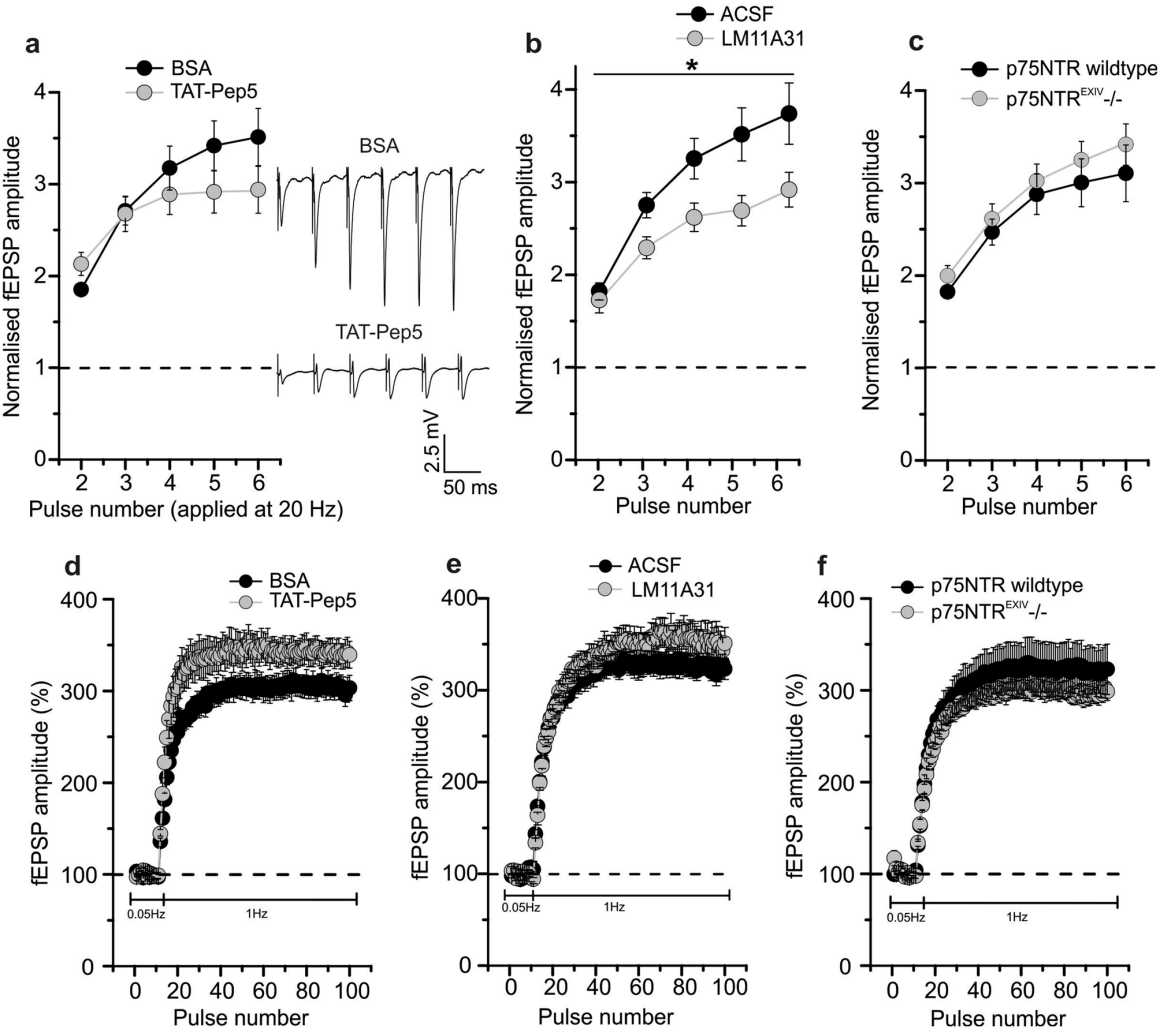
Effect of acute or chronic inhibition of p75NTR signaling on longer lasting short-term synaptic plasticity properties at hippocampal mossy fiber-CA3 synapses. (**a**) and (**b**) extended paired-pulse facilitation paradigm (i.e. train facilitation at 20 Hz) in the presence of bath applied 1 µM TAT-Pep5 (**a**) and 100 nM LM11A31 (**b**) compared to solvent controls. Representative averaged original responses are exhibited for train facilitation in presence of BSA and TAT-Pep5 in the ACSF. Scale bars are displayed in the inset. (**c**) Train facilitation in p75NTR^EXIV−/−^ mice compared to wildtype littermates. (**d**) and (**e**) frequency facilitation at 1 Hz in presence of TAT-Pep5 (**d**) and LM11A31 (**e**). (**f**) Frequency facilitation in p75NTR^EXIV−/−^ mice. Data displayed as mean ± SEM. *p < 0.05 (ANOVA, Mann–Whitney U-test or two-tailed Student’s t-test).

Next, we analyzed the effects of p75NTR signaling on train facilitation (TF), which is another measure of short-term presynaptic plasticity at MF synapses. We hypothesized that the weak p75NTR effects on PPF might become more evident by prolonged stimulation at the same inter-pulse interval. However, TF exhibited no change in the presence of TAT-Pep5 compared to BSA control (Fig. 5a; TF at pulse 4: BSA: 3.2 ± 0.24 (n = 10/N = 4), TAT-Pep5: 2.9 ± 0.22 (n = 11/N = 9), One-way ANOVA F_(1,95)_ = 2.664 p = 0.1059). In the presence of LM11A31, TF was significantly decreased (Fig. 5b; TF at pulse 5: Ctrl: 3.5 ± 0.29 (n = 16/N = 11), LM11A31: 2.7 ± 0.16 (n = 13/N = 8), One-way ANOVA F_(1,135)_ = 18.8 p < 0.0001). Bonferroni post-tests revealed that LM11A31 treated slices displayed significantly decreased TF ratios at pulse 5 (P5; p = 0.0274) and P6 (p = 0.0284), while ratios at P2, P3 and P4 showed no significant change. The p75NTR^EXIV−/−^ animals did not show any difference in TF in comparison to their wildtype littermates (Fig. 5c; TF at pulse 4: WT: 2.9 ± 0.22 (n = 10/N = 5), p75NTR^EXIV−/−^: 3.0 ± 0.18 (n = 18/N = 7), One-way ANOVA F_(1,120)_ = 2.699 p = 0.1030).

Thus, consistent with its effect on PPF (compare Fig. 4), LM11A31 also reduced TF (prolonged stimulation compared to PPF stimulus) at MF synapses, whereas neither acute nor chronic inhibition of p75NTR signaling affected TF. This suggests that modulation of p75NTR by LM11A31 can reduce this type of presynaptic short-term plasticity at MF-CA3 synapses.

In the last approach, we determined whether MF-specific frequency facilitation is dependent on p75NTR signaling. TAT-Pep5 application (1 µM in 0.1% BSA) did not significantly alter frequency facilitation compared to BSA-treated slices (Fig. 5d; BSA: 304.9 ± 12.9% (n = 10/N = 4), TAT-Pep5: 339.6 ± 14.4% (n = 11/N = 10), Mann–Whitney U-test U = 29.0, df = 1, p = 0.067). Similarly, we observed no significant change in frequency facilitation in the presence of LM11A31 and in p75NTR^EXIV−/−^ mice compared to their respective controls (Fig. 5e: Ctrl: 322.1 ± 10.1% (n = 16/N = 11), LM11A31: 349.5 ± 17.2% (n = 13/N = 8), two-tailed Student’s t-test t_(27)_ = − 1.4331 p = 0.1633; Fig. 5f: WT: 321.5 ± 26.3% (n = 9/N = 5), p75NTR^EXIV−/−^: 298.7 ± 9.7% (n = 18/N = 7), Mann–Whitney U-test U = 86.0, df = 1, p = 0.797). Overall, this indicates that neither acute nor chronic interference with p75NTR signaling influence frequency facilitation at hippocampal MF synapses.

Taken together our data suggest that LTD at hippocampal MF synapses seems to involve BDNF, whereas MF LTD is not dependent on p75NTR signaling. Furthermore, we describe novel effects of p75NTR signaling on basal synaptic transmission and short-term synaptic plasticity.

## Discussion

In the hippocampal Cornu Ammonis (CA) 3 region, mossy fiber (MF) and associational/commissural (A/C) synapses possess distinct electrophysiological and anatomical properties^24^. Since little is known about the mechanisms of long-term depression (LTD) in this part of the trisynaptic hippocampal pathway, we focused on low frequency stimulation (LFS) induced LTD at MF-CA3 and A/C-CA3 synapses using the same slice preparation and recording conditions. Our results illustrate that MF-CA3 synapses show efficient LTD that was impaired by acute inhibition of plasminogen activator inhibitor-1 (PAI-1), a manipulation that leads to more efficient conversion of proBDNF to mBDNF. This suggests an involvement of BDNF in MF-LTD. However, MF-LTD was unaltered by either acute or chronic interference with the p75NTR pathway.

To obtain clear results for p75NTR-dependent modulation of MF-LTD, we first had to establish conditions, which allowed us to successfully distinguish recordings where the majority of stimulated axons originated from either MF or AC fiber inputs. A/C synapses could be clearly distinguished from MF synapses by twofold smaller paired-pulse facilitation (PPF), threefold smaller train facilitation (TF), and threefold smaller frequency facilitation, being consistent with previous studies^34,47^ (Fig. S1). In conjunction with the MF specific block of synapses with DCG-IV, we established recording conditions where > 80% of synaptic inputs originated from MFs (see Fig. 3a). We observed similar magnitudes of LTD at MF and A/C fiber inputs when using the identical LFS protocol, which is in agreement with earlier work^48–51^ (compare Fig. 1a). Importantly, HFS induced long-term potentiation (LTP) and LTD at AC-CA3 synapses induced either by low frequency tetanic stimulation (3 Hz, 3 min) or by in vivo 1 Hz LFS were shown previously to critically dependent on NMDAR activation^48,52–55^. Our results demonstrating independence of LFS induced LTD at MF-CA3 synapses from NMDARs (compare Fig. S2), are in line with earlier studies on MF-LTD^56,57^ and further support that our recording conditions (Fig. 1 and Fig. S1) and sorting criteria (compare Materials and Methods) allowed us to pinpoint MF inputs. Since MF-LTP^34^ and MF-LTD (this study) occur both independent of NMDAR signaling, and have previously been described to rely on presynaptic changes^55,58^, these findings indicate that MF and A/C fiber synapses onto CA3 neurons employ distinctly different signaling cascades for LTP and LTD, demonstrating the unique NMDAR-independent long-term synaptic plasticity of MF synapses.

The role of proBDNF/p75NTR signaling in LTD has been studied in detail at hippocampal Schaffer collateral (SC)-CA1 synapses^17,18,46,59^, whereas a role of proBDNF in LTD at MF-CA3 synapses remained elusive. In the current study, we addressed this question by using either acute or chronic “interference” with proBDNF/p75NTR signaling in LFS induced or paired-pulse (pp) LFS^38,39^ induced MF-LTD. To test for acute BDNF effects, we applied an inhibitor of PAI-1 (tiplaxtinin), which facilitates the conversion of proBDNF to mature BDNF^41^. For acute inhibition of p75NTR signaling, we either applied the p75NTR antagonist TAT-Pep5^36^ or the p75NTR modulator LM11A31^37^. Chronic effects of impaired p75NTR signaling were tested in slices from p75NTR^ExIV^ knock-out (−/−) mice compared to their wildtype littermates^40^. Regarding LM11A31, Yang and coworkers showed that it prevents amyloid-β induced degeneration and synaptic dysfunction through p75NTR signaling^37^. Based on this evidence, we used LM11A31, as a p75NTR ligand, to investigate positive or negative modulation of p75NTR signaling in MF-LTD.

As described in the results (Fig. 1), LTD at hippocampal MF-CA3 synapses displayed significant impairment in the presence of tiplaxtinin in the ACSF (Fig. 1b). Nevertheless, we did not observe any effect of LM11A31 on MF LTD (Fig. 2c). Likewise, also the intracellular p75NTR antagonist TAT-Pep5 did not impair MF-LTD (compare Fig. 2a). Together, these results provide two independent lines of evidence that acute inhibition of p75NTR signaling does not interfere with induction and expression of MF-LTD. In accordance with this observation, also chronic inhibition of p75NTR signaling in p75NTR^ExIV−/−^ mice, in which both, the short and the full-length isoforms of p75NTRs are constitutively deleted^40,60^, did not affect induction or expression of MF-LTD.

Taken together, these results suggest that MF-LTD recruits proBDNF signaling, similar to SC-CA1 LTD, yet this effect is mediated independent of p75NTR signaling.

Our observation of unaltered MF-LTD in the presence of the tyrosine kinase inhibitor K252a, when applied in the same way that inhibits MF-LTP in our hands^34^, as well as the unaltered MF-LTD in the presence of the selective TrkB receptor inhibitor ANA-12^43^ (Fig. 2d,e) provide two independent lines of evidence that also TrkB receptor signaling is not involved in MF-LTD. While this is at variance with earlier studies showing that the group I metabotropic glutamate receptor (mGluR) agonist DHPG (3, 5-dihydroxyphenylglycine) can induce MF-LTD through BDNF release and TrkB receptor activation^61,62^, these previous studies used chemical induction of LTD in juvenile rats, whereas our study employed low frequency electrical stimulation for LTD induction in adult mice. Thus, it seems conceivable that depending on the type of LTD stimulus, the animal species and/or the age of animals, different mechanistically distinct forms of LTD might coexist at hippocampal MF-CA3 synapses (as shown previously e.g. for LTP at SC-CA1 synapses)^20,63,64^.

It could be argued that the inhibited MF-LTD observed in the presence of tiplaxtinin (compare Fig. 1b) might be mediated by tiplaxtinin induced increased extracellular levels of mature BDNF that could reduce MF-LTD through TrkB signaling. Since co-application of ANA-12 and tiplaxtinin induced precipitates in our recording solution, we could not answer this question (data not shown). However, since ANA-12 alone did not change MF-LTD (Fig. 2e), a role of TrkB signaling in MF-LTD under control conditions is not evident.

Similar to its involvement in MF-LTD, the role of proBDNF/p75NTR signaling in basal synaptic transmission and short-term plasticity at MF-CA3 synapses has, to the best of our knowledge, not been reported before. Interestingly, although p75NTR signaling did not play a role in MF-LTD, our data show that it is involved in short-term synaptic plasticity at MF synapses, particularly in PPF and TF. Thus, acute inhibition of p75NTRs with TAT-Pep5 resulted in increased PPF at an inter-stimulus interval (ISI) of 20 ms (Fig. 4d). Consistent with this result for acute inhibition of p75NTRs, chronic inhibition of p75NTR signaling in p75NTR^EXIV−/−^ mice also resulted in increased PPF, specifically at an ISI interval of 20 ms (Fig. 4f). These data suggest a presynaptic modulatory effect only at short ISI (20 ms) PPF stimulation upon acute or chronic inhibition of p75NTR signaling. Importantly, the absence of an effect on input–output (IO) curves, TF, and frequency facilitation after acute inhibition with TAT-Pep5 is in agreement with our observation that also chronic inhibition of p75NTR signaling in p75NTR^EXIV−/−^ mice does not affect these synaptic properties.

Interestingly, the experiments employing acute application of the p75NTR ligand LM11A31 revealed variations with the acute effects of TAT-Pep5 in particular for IO curve, PPF and TF. Thus, we observed a steeper IO curve (Fig. 4b), decreased PPF (Fig. 4e) and significantly reduced TF (Fig. 5b) following application of LM11A31 compared to respective controls. Since we did not see similar effects for the acute p75NTR inhibitor TAT-Pep5, our findings lead us to suggest that in our experiments LM11A31 did not act as a negative regulator of the p75NTR. Whether LM11A31 exerts rather agonistic or modulatory functions on p75NTRs cannot be concluded from our experiments.

Importantly, acute application of tiplaxtinin revealed a parallel p75NTR-independent involvement of BDNF in MF-LTD and in frequency facilitation at MF-CA3 synapses (compare Fig. 1). Interestingly, both observations might be connected. Since frequency facilitation is reduced in the presence of tiplaxtinin and is elicited by the same 1 Hz stimulation (for 100 s) of MF synapses that we used for inducing MF-LTD (LFS at 1 Hz for 15 min), it is tempting to speculate that the decreased amplitudes of MF fEPSPs in the presence of tiplaxtinin during the LTD induction stimulus might reduce the efficiency to elicit MF-LTD. Future studies measuring presynaptic Ca^2+^ levels in MF terminals during induction of LFS induced MF-LTD in the presence of tiplaxtinin might help to pinpoint such a role of proBDNF signaling in the induction process for MF-LTD.

Interestingly, absence of an effect on basal synaptic transmission (IO curves) and TF after acute application of tiplaxtinin is in agreement with our observation that also acute inhibition (TAT-Pep5) and chronic inhibition of p75NTR signaling in p75NTR^EXIV−/−^ mice does not affect these synaptic properties. Given the high expression of BDNF protein in MFs^67^ and the characteristic dependence of BDNF release on sustained synaptic stimulation (compare recent reviews^3,19^) this could be taken to indicate proBDNF and mature BDNF release during the maintained 1 Hz stimulation used to obtain frequency facilitation and LTD at MF synapses. In contrast, proBDNF/mBDNF cannot be released by less synaptic stimulation, explaining why basal synaptic properties and TF are not affected by these rhythms of stimulation—neither by BDNF acting through p75NTR nor through p75NTR-independent pathways.

In conclusion, we show here for the first time that LFS or ppLFS induced LTD at mouse hippocampal MF-CA3 synapses is not impaired or modified after both, chronic p75NTR deletion or acute inhibition of p75NTR signaling. Moreover, MF-LTD does also not seem to involve TrkB receptor signaling. Nonetheless, MF-LTD is significantly impaired by acute inhibition of PAI-1, an inhibitor of both extracellular and intracellular proBDNF cleavage, a manipulation that leads to more effective conversion of proBDNF to mBDNF. In addition, we observed a yet not described effect of acute inhibition of p75NTR signaling on short-term plasticity (PPR). Since MF-CA3 synapses play a crucial role in pattern separation and pattern completion and thereby in memory formation^49,65^, our results seem to suggest the interesting possibility that proBDNF via non-canonical, yet unknown signaling could be important for information processing in the hippocampus. Altogether, our data highlight that basal and activity-dependent glutamatergic transmission at MF-CA3 synapses depends on unconventional signaling mechanisms compared to SC-CA1 synapses.

## Materials and methods

### Animals

Long-term depression (LTD) experiments were performed on 400 µm thick acute transversal hippocampal slices from 8 to 14 weeks old male C57Bl/6J mice (Charles River, Sulzfeld, Germany; local distributor of Jackson Lab.), or p75NTR^ExIV^ knock-out (−/−) mice^40^ and their respective wildtype littermates. Genotypes of p75NTR^EXIV−/−^ and their WT littermates were determined by PCR (primer sequence: 5′-GAT GGA TCA CAA GGT CTA CGC-3′) from ear punches. Mice were randomized for treatments. Experiments were performed independently by 2 investigators (except for experiments employing LM11A31)—one of which was blinded to the identity of the pharmacological manipulations. In experiments with p75^NTR^ ko mice, the experimenter was blinded for the genotype of the animals. All experimental procedures were carried out following the ethical guidelines for the use of animals in experiments, and were conducted in compliance with the European Committees Council Directive on animal experimentation (2010/63/EU) and the ARRIVE guidelines. All experiments were approved by the Local Animal Care Committee of the state Saxony-Anhalt (Landesverwaltungsamt Saxony-Anhalt, Germany, No. 42502-2-1191 UniMD).

### Hippocampal slice preparation and maintenance

The animals were decapitated after being anesthetized with isoflurane (Isofluran CP, cp-pharma, Germany). The brain was removed immediately and kept in the ice-cold carboxygenated artificial cerebrospinal fluid (ACSF in mM: 125 NaCl, 2.5 KCl, 26 NaHCO_3_, 0.8 NaH_2_PO_4_, 6 MgCl_2_, 1 CaCl_2_, 10 glucose, saturated with 95% O_2_ and 5% CO_2_). The brain was cut in ice-cold carboxygenated ACSF with a vibratome (Pelco vibratome Series 1000, Technical Products International Inc., USA). Transversal hippocampal slices were incubated in carboxygenated ACSF at 34 °C for 25 min followed by 1 h recovery at 30–32 °C in interface-style chamber before the start of the experiment.

### Field EPSP measurements

Field potential measurements were carried out in interface-style chamber with continuous perfusion (1.5 ml/min) of carboxygenated ACSF (in mM: 125 NaCl, 2.5 KCl, 26 NaHCO_3_, 0.8 NaH_2_PO_4_, 4 MgCl_2_, 4 CaCl_2_ and 10 glucose). In order to prevent polysynaptic signal at mossy fiber (MF)–Cornu Ammonis (CA) 3 synapses, the Ca^2+^/Mg^2+^ ratio was set to 4:4^26, 34^. The temperature of the external solution in the interface chamber was kept at 32 °C with a temperature controller (FST TR-100, Fine Science Tools, Germany). Field potential responses were induced with a concentric bipolar stimulation electrode (Frederick Hear & Co., Bowdoin, USA) by stimulating the granule cell layer of the dentate gyrus. A glass electrode (~ 4–6 MΩ) filled with ACSF was placed in stratum lucidum of CA3 to record MF field potentials. Same type of stimulation and recording electrodes were used for associational/ commissural (A/C) responses. The stimulation electrode was placed in the stratum radiatum of CA3 region, nearby dentate gyrus and A/C field potentials were recorded by placement of a recording electrode in stratum radiatum of CA3 region, proximal to CA2 region (see schematic in Fig. 1). The acute hippocampal slice preparation, recording conditions and stimulus paradigms used for field EPSP recording at Schaffer collateral (SC)-CA1 synapses were similar to MF-CA3 synapses. However, the stimulation electrode was placed between CA3 and CA1 region at the location of the Schaffer collaterals and SC field potentials were recorded by placement of a recording electrode in stratum radiatum of CA1 region. An Ext-02F/2 amplifier (npi, Germany) and a CED 1401 interface were used for extracellular field potential measurements and all data were filtered at 10 kHz. The gain was set to 1000. The field potential (fEPSP) amplitude was calculated with the help of the Intracell program (Leibniz Institute of Neurobiology Magdeburg, Germany) between fEPSP onset and maximal amplitude.

Basal synaptic transmission was assessed with the help of an input–output (IO; 0–400 µA for 0.1 ms, 20 or 50 µA increments) curve and paired-pulse facilitation (PPF). From the IO curve, half maximal stimulation was chosen for subsequent experiments. The PPF was tested at 20, 50, 100 and 200 ms inter-stimulus intervals (ISI), and averaged over 3 stimulations at 0.05 Hz. The MF signals were distinguished from A/C signals by their distinct properties with respect to paired-pulse ratio (PPR), train facilitation (6 pulses with 50 ms interpulse interval, repeated 3 × at 0.05 Hz) and frequency facilitation (10 pulses at 0.05 Hz (baseline), subsequently followed by 90 pulses at 1 Hz). Field EPSPs had to meet the following criteria to be identified as pure MF signals:

1. Train facilitation of at least 250% at the end of the train
2. Frequency facilitation of at least 250%
3. Reduction of fEPSP amplitudes to 20% in response to bath application of the group II mGluR agonist (2S,2′R,3′R)-2-(2′,3′-dicarboxycyclopropyl)-glycin (DCG-IV, 1 μM, Tocris, USA).

The field potentials were identified as A/C signals when both train facilitation and frequency facilitation of < 150% was observed^34^. Field EPSPs which demonstrated either train facilitation or frequency facilitation in-between ratios (150–250%) were excluded from data analyses. We tested IO curve, PPF, train facilitation and frequency facilitation in all slices before inducing LTD. After determining train facilitation and frequency facilitation, we waited for 2–5 min to allow MF and A/C fEPSPs to recover to the same baseline level as before inducing facilitation. Afterwards, baseline recording was performed for 10 min, and low frequency stimulation (LFS, 900 pulses at 1 Hz)^48^ or paired-pulse LFS (900 pulses at 1 Hz; inter-pulse interval 50 ms) ^38, 39^ was used to induce LTD. Field potentials were recorded for 60 min after LTD induction. MF origin of fEPSPs were confirmed with DCG-IV^66^ application at the end of recording (Figs. 1, 2 and 3).

To confirm the MF origin of fEPSPs under our recording conditions, we also performed a separate set of experiments with DCG-IV application only (i.e., continuous recordings without LTD induction). Following 10 min baseline recording (i.e. synaptic stimulation at 0.05 Hz), DCG-IV was applied and the MF fEPSP amplitude was monitored 10 min after start of application.

To validate N-methyl D-aspartate receptor (NMDAR) independency of LTD and short-term plasticity at MF-CA3 synapses, the NMDAR antagonist APV (50 µM dissolved in water, DL-2-Amino-5-phosphonopentanoic acid, Tocris, Germany) was used. To test for acute effects of BDNF signaling via tropomyosin related kinase (Trk) B, the tyrosine kinase inhibitor K252a (200 nM, Alomone Labs; Israel; dissolved in 0.1% Dimethyl sulfoxide (DMSO; Sigma Aldrich, Germany) was bath applied. In another approach, bath application of ANA-12 (20 µM in 0.1% DMSO)^43^ was used to selectively inhibit TrkB receptor signaling. To test for effects of acute inhibition of BDNF signaling via p75 neuroptrophin receptors (p75NTR) on MF-LTD, the p75NTR ligand LM11A31^37^ (100 nM, dissolved in water; R&D Systems, Germany) was used. In another series of experiments, bath application of TAT-Pep5^36^ (1 µM in 0.1% Bovine Serum Albumin (BSA); Calbiochem) was used to inhibit p75NTR signaling. ProBDNF signaling effects on MF-LTD were tested with acute application of tiplaxtinin (150 µM in 0.1% DMSO; Selleckchem), an inhibitor of plasminogen activator inhibitor-1^41^. MF-LTD experiments with ANA-12, tiplaxtinin and respective DMSO controls (100–200 ml ACSF) were performed with a pump-driven recycling perfusion system^34^. For the LTD experiments with TAT-Pep5, tiplaxtinin and respective controls, we used a micro-perfusion pump-driven solution recycling system (Bioptechs Delta T-Micro-Perfusion pump high flow, ChromaPhor, Germany) with a total ACSF volume of 50 ml. To reduce unspecific binding of TAT-Pep5, perfusion tubings were coated with 0.1% BSA prior use. Respective vehicle solution (0.1% DMSO, 0.1% BSA) incubated slices were used as positive controls under the same perfusion system conditions. All drugs were bath applied (perfusion rate: 1.5 ml/min) and slices were pre-incubated for ≥ 1 h with inhibitors before start of recording. Chronic impairment of p75NTR signaling was assessed in p75NTR^EXIV−/−^ mice and their respective wildtype littermates. All experimental conditions remained the same, aside from the absence of any blockers.

### Data analysis and statistics

The PPR was determined by calculating the change in mean response size of the 2nd divided by the 1st fEPSP. Train facilitation was calculated as the fEPSP amplitudes of 2nd to 6th pulse, normalized to the mean amplitude of the 1st fEPSP. For frequency facilitation assessment, the field potential amplitudes were normalized to the mean baseline of the first 10 pulses (0.05 Hz stimulation), which was then set to 100%. Frequency facilitation was quantified as the normalized change in mean response size during the last 5 pulses delivered at 1 Hz. In the control and LTD measurements, fEPSP amplitudes were normalized to mean baseline between − 10 and 0 min, which was then set to 100%. In all experiments, LTD was determined as the normalized change in mean response size during the last 5 min of measurement (55–60 min after LTD induction) compared to baseline.

The stability of recording conditions was assessed by continuous MF fEPSP recording for over 90 min, with no LTD induction. To assess the impact of short-term plasticity paradigms on basal field potentials, baseline recording was performed for 10 min (MF synapses) or 10 pulses at 0.05 Hz for AC-CA3 synapses, followed by execution of short-term plasticity paradigms, i.e. PPF, train facilitation and frequency facilitation in acute slices. Afterwards, we waited for 2–5 min to allow MF and A/C fEPSPs to recover and baseline was recorded for next 10 min. fEPSP amplitudes were normalized to mean baseline between − 10 and 0 min, which was then set to 100%. Here, synaptic change was determined as the normalized change in mean response size during the last 5 min of measurement compared to baseline.

Statistical analyses were carried out using Origin, GraphPad Prism and MYSTAT programs. Pooled data of experiments from at least 3 different animals were expressed as mean ± standard error of mean (SEM). Furthermore, the number of experiments (n) and number of animals (N) are indicated in the results.

Distribution of data was analyzed with Shapiro–Wilk test. Comparison between controls and test experiments was performed with two-tailed Student’s t-test or Mann–Whitney U-test for parametric or nonparametric data, respectively. One-way ANOVA was used for multiple comparisons. In case of significant main effects, the ANOVA was followed by Bonferroni adaptation for multiple comparisons. A p value of < 0.05 was set as level of significance and indicated by *p < 0.05. The actual statistical procedure used for each experiment is mentioned in the respective text passages.

## Supplementary Information

Supplementary Information.

## Data Availability

The datasets generated during and/or analyzed during the current study are available from the corresponding author on reasonable request.
