# Supplementary material for: Long-term depression at hippocampal mossy fiber-CA3 synapses involves BDNF but is not mediated by p75NTR signaling
Source: Sci Rep. 2021 Apr 20;11:8535. doi: 10.1038/s41598-021-87769-9 (PMC8058084; doi:10.1038/s41598-021-87769-9)
Supplement: Supplementary file 1 — Supplementary Information. [file 41598_2021_87769_MOESM1_ESM.pdf]

Long-term depression at hippocampal mossy fiber-CA3 synapses involves BDNF but is not mediated by p75NTR signaling

Machhindra Garad, Elke Edelmann, Volkmar Leßmann

Supplementary Results and Figures

Mossy fiber (MF)-CA3 and Associational/Commisural fiber (A/C)-CA3 synaptic responses can be clearly distinguished under our recording conditions

We compared field excitatory postsynaptic potentials (fEPSPs) elicited by stimulation of A/C fibers with fEPSPs originating from mossy fibers. A/C-CA3 fEPSPs exhibited significantly steeper input-output (IO) curves (**Fig. S1a**, half maximal stimulation intensity; 160 µA: MF: 0.9 ± 0.10 mV (n=18 / N=14), A/C: 1.6 ± 0.29 mV (n=14 / N=10), One-way ANOVA $F_{(1,450)}$=54.10 p<0.0001). Moreover, we observed significantly higher magnitudes of paired-pulse facilitation (PPF) for all tested inter-stimulus intervals (ISIs; 20, 50, 100 and 200 ms) at MF compared to A/C fiber synapses (**Fig. S1b**, PPF at ISI 200 ms: MF: 1.6 ± 0.05 (n=18 / N=14), A/C: 0.9 ± 0.05 (n=14 / N=10), One-way ANOVA $F_{(1,120)}$=66.90 p<0.0001). In addition, the train facilitation (TF) was significantly higher at MF-CA3 synapses compared to A/C fiber synapses (**Fig. S1c**, TF at pulse 2: MF: 2.1 ± 0.06 (n=18 / N=14), A/C: 1.2 ± 0.05 (n=14 / N=10), One-way ANOVA $F_{(1,155)}$=233.6 p<0.0001). Furthermore, MF-CA3 synapses displayed significantly increased frequency facilitation compared to A/C fiber synapses (**Fig. S1d**; MF: 314.0 ± 10.9 % (n=18 / N=14), A/C: 122.4 ± 6.1 % (n=14 / N=10), two-tailed Student's t-test: $t_{(30)}$= 14.0951 p<0.0001). Finally, application of 1 µM of the group 2 mGluR agonist, (2S,2′R,3′R)-2-(2′,3′-dicarboxycyclo-propyl)glycine (DCG-IV) robustly blocked the MF field potentials (**Fig. S5c**; Ctrl: 99.2 ± 0.8%, DCG-IV: 19.9 ± 4.2% (n=10 / N=3), paired Student's t-test: $t_{(9)}$=18.7948 p<0.0001). These results indicate that under our recording conditions we can differentiate pure MF signals from A/C fiber signals.

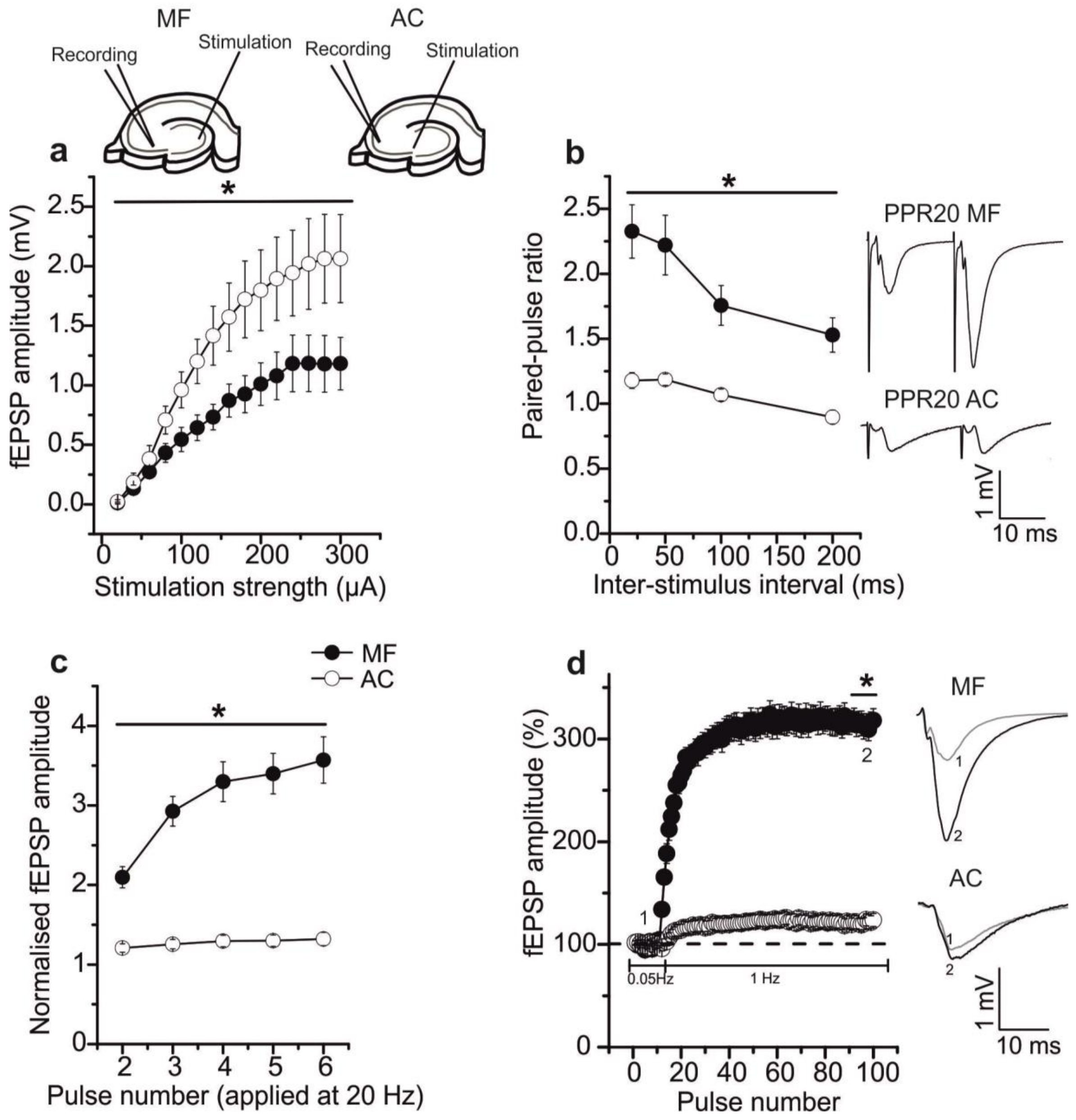

Figure S1: *Mossy fiber (MF) and associational/commissural (A/C) fibers displayed different basal synaptic transmission and short-term plasticity.* Schematic shows stimulation and recording sites for MF-CA3 and A/C-CA3 fEPSPs. **a)** Input-output curve of basal synaptic responses of pure MF synapses was less steep than at A/C fiber synapses. **b)** Paired-pulse facilitation (PPF) at inter-stimulus intervals (ISI) of 20, 50, 100 and 200 ms was significantly higher at MF than at A/C fiber synapses. Representative averaged original responses are shown for PPF at an ISI of 20 ms for MF and A/C fibers. **c)** Extended PPF paradigm, termed train facilitation at 20 Hz stimulation revealed significantly higher values at MF synapses in comparison to A/C fiber

synapses. **d**) MF-CA3 synapses exhibited significantly higher frequency facilitation at 1 Hz compared to A/C fibers (-●-: MF (n=18 / N=14); -○-: A/C fibers (n=14 / N=10)). Representative mean original traces are shown for both groups. In the figure, “1” depicts mean fEPSP amplitudes of the first 10 responses at 0.05Hz, and “2” indicates mean fEPSP amplitudes of the last 5 responses of 90 pulses at 1 Hz.

MF-CA3 LTD is not dependent on NMDA receptor activation

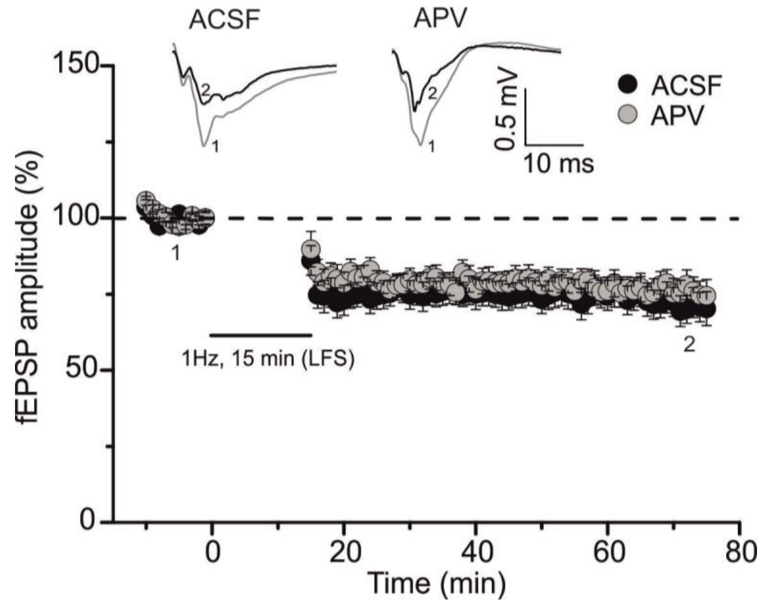

Figure S2**:** *MF-CA3 LTD is not induced through activation of NMDA receptors.* MF synapses displayed similar LTD magnitude in presence of APV (50 µM; NMDAR inhibitor) compared to control (●: ACSF (n=16 / N=12); ○: APV (n=17 / N=13)). The inset shows representative averaged original fEPSP responses. For MF-LTD in normal ACSF and in the presence of APV, “1” depicts mean fEPSP amplitudes of first 10 min of baseline and “2” depict mean fEPSP amplitudes between 55-60 min after induction of LTD. Data are expressed as mean ± SEM. Corresponding scale bars are shown in the respective insets. *: $p<0.05$ (ANOVA or two-tailed Student’s t-test).

LTD at Schaffer collateral-CA1 synapses is impaired in the presence of the p75NTR antagonist TAT-Pep5

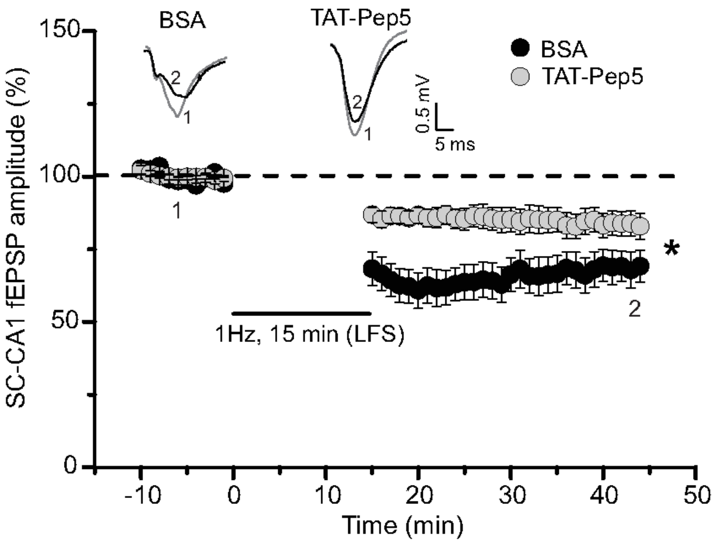

Figure S3: *Acute inhibition of p75NTR signaling with 1 µM TAT-Pep5 significantly impairs LTD at hippocampal Schaffer collateral (SC)-CA1 synapses.* LTD at SC-CA1 synapses, induced by low frequency stimulation (LFS, 1 Hz, 15 min), was significantly impaired in presence of TAT-Pep5 compared to BSA control (●: BSA (n=12 / N=8); ○: TAT-Pep5 (n=11 / N=7)). Representative averaged original responses are shown for BSA and TAT-Pep5 treated slices. In the graph, "1" depicts mean fEPSP amplitudes of first 10 min of baseline and "2" indicates mean fEPSP amplitudes between 25-30 min after induction of LTD. Data shown as mean ± SEM. Scale bars are exhibited in the inset. *: $p<0.05$ (two-tailed Student's t-test)

Mossy Fiber (MF)-CA3 synapses show intact short-term plasticity upon acute inhibition of NMDA receptors

In the presence of APV, we observed a slightly steeper input-output (IO) curve at MF-CA3 synapses compared to ACSF control (**Fig. S4a**; half maximal stimulation intensity; 200 µA: ACSF: 1.0 ± 0.18 mV (n=16 / N=12), APV: 1.4 ± 0.23 mV (n=12 / N=8), ANOVA $F_{(1,407)}$=19.74

p<0.0001). Nonetheless, APV treated slices displayed intact paired-pulse facilitation (PPF) at inter-stimulus interval (ISI) of 20, 50, 100 and 200 ms (**Fig. S4b**; PPF at ISI 20 ms: ACSF: 2.3 ± 0.20 (n=16 / N=12), APV: 2.2 ± 0.18 (n=12 / N=8), ANOVA $F_{(1,100)}$=2.633 p=0.1078), train facilitation (TF) (**Fig. S4c**; TF at pulse 4: ACSF: 3.3 ± 0.25 (n=16 / N=12), APV: 3.6 ± 0.33 (n=12 / N=8), ANOVA $F_{(1,130)}$=1.172 p=0.2810). In addition, MF-CA3 synapses in the hippocampus exhibited normal frequency facilitation in presence of APV (**Fig. S4d**; ACSF: 314.4 ± 12.2 % (n=16/N=12); APV: 289.8 ± 11.0 % (n=12/N=8), Mann-Whitney U-test U=113.0 df=1 p=0.082). Altogether, these results indicate that presence of APV, an NMDA receptor antagonist, though affects IO curve slightly; it does not impact short-term plasticity (PPF, TF and frequency facilitation) at MF-CA3 synapses.

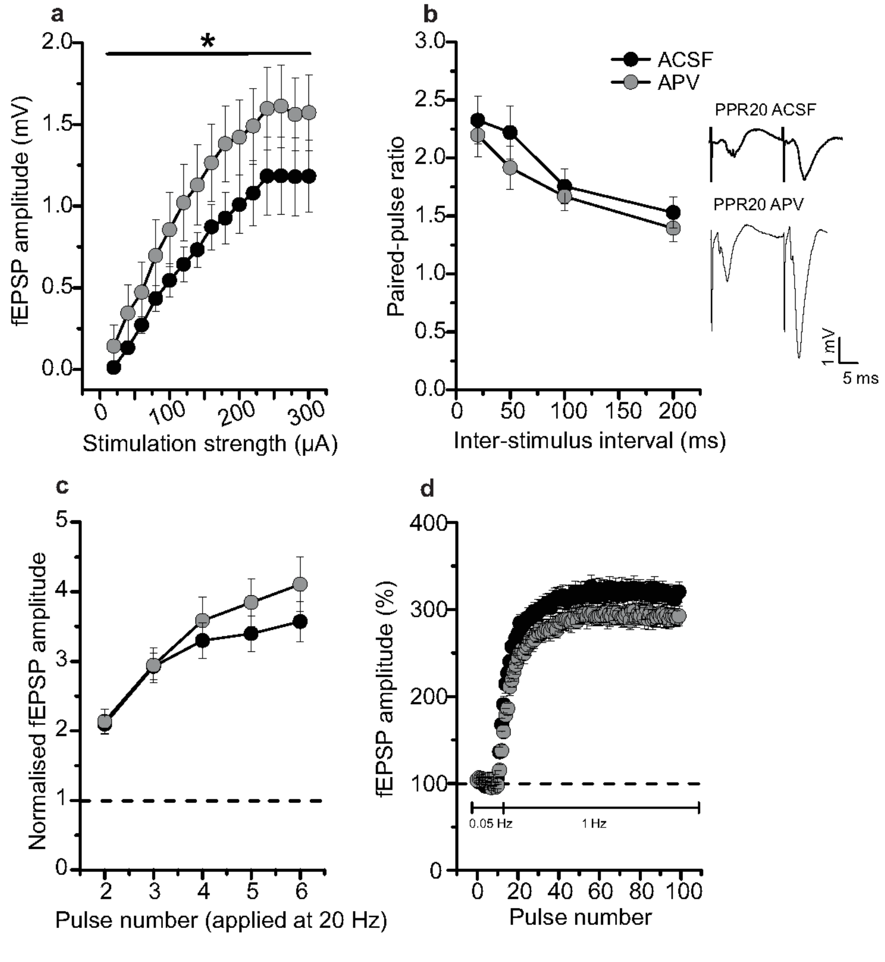

Figure S4: *Influence of acute inhibition of NMDA receptors on basal synaptic responses and short-term synaptic plasticity at hippocampal mossy fiber synapses.* ●: ACSF (n=16 / N=12); ○: APV (n=12 / N=8). **a)** and **b)** input-output curve and paired-pulse facilitation (PPF) at different inter-stimulus intervals (ISI) of (20, 50, 100 and 200 ms) at MF-CA3 synapses in the presence of 50 µM APV, for acute inhibition of NMDA receptors. **c)** and **d)** Train facilitation and frequency facilitation (1 Hz) at hippocampal MF-CA3 synapses in APV treated slices in comparison to ACSF control. The inset displays representative averaged original responses for PPF at an ISI of 20 ms for ACSF and APV. Corresponding scale bars are shown in the inset. Data expressed as mean ± SEM. *: $p<0.05$ (ANOVA or Mann-Whitney U-test)

Mossy fiber (MF)-CA3 and A/C-CA3 synaptic responses are not affected by short-term synaptic plasticity protocols and remain stable for at least 90 min

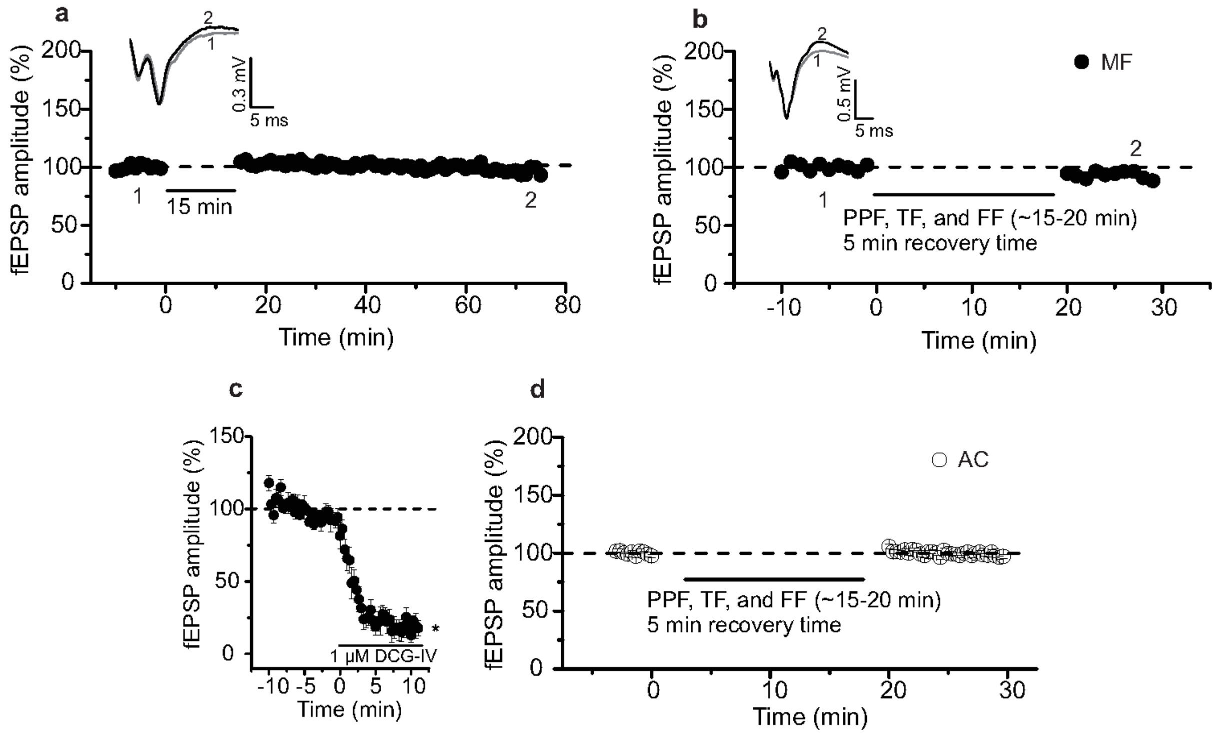

Figure S5: *Stable MF-CA3 fEPSP over 90 min. MF and A/C synapses show no influence of short-term plasticity paradigms on basal fEPSP responses.* **a)** Averaged MF fEPSP with no LTD

induction reveals stable fEPSP amplitudes over 90 min of recording (●: MF (n=9 / N=6)). Re-presentative averaged original responses are shown in the inset. In the inset, “1” depicts mean fEPSP amplitudes of first 10 min of baseline and “2” indicates mean fEPSP amplitudes between 70-75 min. **b)** Stable MF fEPSP amplitudes followed by 2-5 min recovery after short-term plasticity paradigms (●: MF (n=12 / N=7)). In the inset, “1” depicts mean fEPSP amplitudes of first 10 min of baseline and “2” indicates mean fEPSP amplitudes between 25-30 min. Scale bars are shown in the respective insets. **c)** In a separate set of experiments, pure MF origin of signals was confirmed by reduction of fEPSP amplitudes in response to bath application of 1 µM DCG-IV to 19.9 ± 4.2% (n=10 / N=3). **d)** Stable A/C-CA3 fEPSP amplitudes followed by 2-5 min recovery after short-term plasticity including frequency facilitation paradigms (○: A/C (n=10 / N=7)). Data shown as mean ± SEM. *: $p<0.05$ (1-sample Student’s t-test or paired Student’s t-test)
